# Evaluation of the diagnostic performance of a commercial ELISA based on two recombinant antigens for the diagnosis of *Strongyloides stercoralis* infection

**DOI:** 10.1186/s13071-026-07542-7

**Published:** 2026-07-04

**Authors:** Ángel Gustavo Guevara, Yosselin Vicuña, Mariella Anselmi, Monica Marquez, Francisco Robinzón Huerlo, Matteo Valerio, Cristina Mazzi, Francesca Tamarozzi, Dora Buonfrate

**Affiliations:** 1 Instituto de Investigación en Biomedicina, Carrera de Medicina, Universidad Central del Ecuador (UCE), Quito, Ecuador; 2 Centro de Epidemiologia Comunitaria y Medicina Tropical (CECOMET), Esmeraldas, Ecuador; 3 Centro Especialidades Médicas Madre Anastasia, Esmeraldas, Ecuador; 4 Biostatistic Unit, IRCCS Sacro Cuore Don Calabria Hospital, Negrar di Valpolicella, VR Italy; 5 Department of Infectious Tropical Diseases and Microbiology, IRCCS Sacro Cuore Don Calabria Hospital, Negrar di Valpolicella, VR Italy

**Keywords:** *Strongyloides stercoralis*, Control programmes, ELISA, Recombinant antigen, Target product profile

## Abstract

**Background:**

The WHO guideline for the public health control of *Strongyloides stercoralis* recommends the deployment of Baermann or agar plate culture to estimate the infection prevalence. However, the guideline includes the possible use of antibody-based assays, which might be preferred in some countries. An ELISA based on two recombinant antigens (Strongy Detect ELISA by InBios), performed on dried blood spots (DBS), demonstrated suitable for use in a diagnostic study carried out in remote areas of Ecuador (“ESTRELLA”). A new version of that assay, which aimed at improving specificity according to the indications reported in the target product profile (TPP) for the diagnostics for *S. stercoralis*, was recently made available in the market. Aim of this study was to compare the performance of the two versions of that ELISA.

**Methods:**

The DBS collected in the context of the study in Ecuador were tested with the new ELISA. Sensitivity, specificity, positive and negative predictive values (PPV and NPV, respectively) were calculated with Bayesian Latent Class Analysis (BLCA). Bayesian estimates were reported as posterior medians with corresponding 95% credible intervals (CrI). Test agreement was calculated with both BLCA and Cohen’s kappa.

**Results:**

Sensitivity was 66.7% (95% CrI 52.8–80.6) for the new assay, versus 78.8% (95% CrI 66.7–89.2) of the old version (posterior probability that the new test had higher sensitivity = 0.1). Specificity was markedly higher for the new version compared to the previous assay: 98.8% (95% CrI 97.9–99.4) versus 90.1% (95% CrI 87.6–92.4), respectively (posterior probability that the new test had higher specificity = 1). PPV for the new ELISA proved good at all prevalence values considered, going from 80.1% (CrI 67.0–89.4%) when prevalence was 7% to 95.8% (92.5%, 97.9%) with 30% prevalence. The agreement was fair (Cohen’s kappa 0.38; 95% CI 0.26–0.49).

**Conclusions:**

The new version of the Strongy Detect ELISA demonstrated improved specificity with a moderate reduction in sensitivity compared to the previous version, meeting the TPP requirements in terms of diagnostic performance. Adherence with the other TPP requirements was out of the scope of this study, and should be assessed elsewhere.

**Graphical Abstract:**

**Figure:**
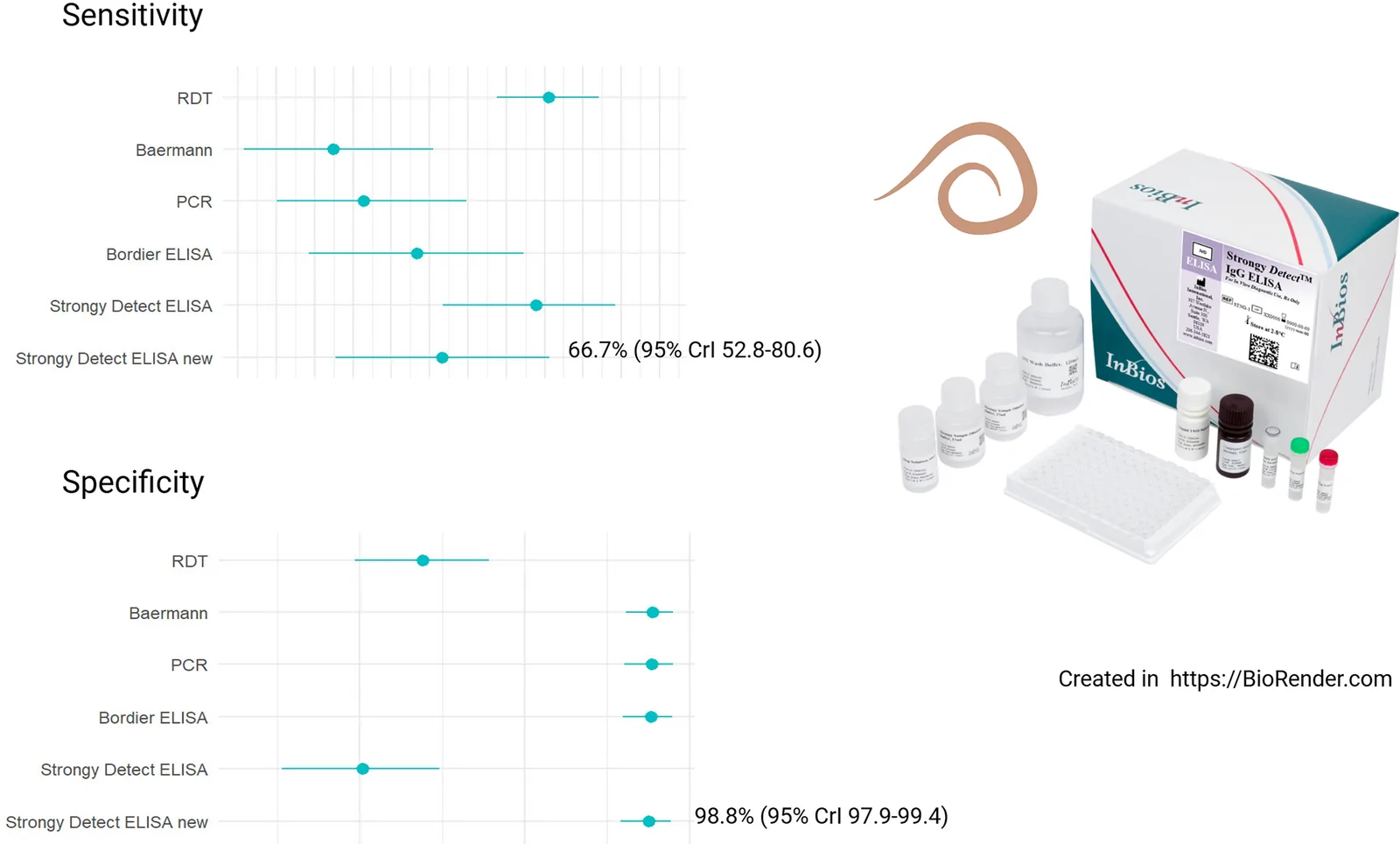

*Strongyloides stercoralis* is a soil-transmitted helminth (STH) which causes strongyloidiasis, a condition listed as one of the neglected tropical diseases (NTDs) by the World Health Organization (WHO) [1, 2]. The infection has been estimated to affect between 300 and 600 million people globally, mostly distributed in disadvantages areas of the world [3–5].

Due to a peculiar auto-infective cycle, human strongyloidiasis can perpetuate indefinitely in the infected host, contrarily to the other STH. Chronic infection can be mostly silent, or cause a wide range of unspecific symptoms, which can have different grades of severity. In particular, the immunosuppressed host is at risk of developing the life-threatening form of disease, hyperinfection or dissemination [6].

Due to the substantial burden caused, the WHO has issued guideline for the control of strongyloidiasis in endemic countries. Main recommendation is to implement mass drug administration (MDA) with ivermectin in all individuals above 5 years of age in areas where *S. stercoralis* prevalence is found ≥ 5% in a survey carried out with agar plate culture or Baermann method [1, 2]. However, these tests can be cumbersome, require fresh feces, and good parasitology skills to differentiate *S. stercoralis* larvae from those of other parasites [7–9]. Hence, the guideline includes the possibility of using antibody assays, which can be of easier implementation in some settings.

The WHO does not specify which serology assays should be used for this purpose. However, high specificity rather than high sensitivity has shown to be critical for a diagnostic test to be used for a control programme for *S. stercoralis,* as reported in the target product profile (TPP) released by the WHO [10, 11], and aimed at defining the minimum and ideal characteristics of a test to be used for public health purpose. Serology assays can be based either on crude or recombinant antigens; theoretically, the latter are supposed to have higher specificity at the expenses of lower sensitivity, with the advantage over the former of high reproducibility and scalability [12]. However, in a diagnostic study carried out in Ecuador (“ESTRELLA”) [13], a commercial ELISA based on *Strongyloides ratti* demonstrated higher specificity (100.0%, 95% credible intervals [CrI] 99.8–100.00) compared to an antibody-detecting rapid test based on the recombinant antigen NIE (93.6%, 95% CrI 91.8–95.3) and a research-use-only (RUO) ELISA based on a combination of two recombinant antigens (91.7%, 95% CrI 89.6–93.8). In ESTRELLA, the ELISAs were performed on dried blood spots (DBS) from finger prick, a procedure that was well accepted by the population under study (school-age children) and that permitted an easy transport of the samples from the field to the central laboratory.

Recently, the RUO ELISA assay used in the ESTRELLA study (Strongy Detect ELISA by InBios) entered in the market [14]. The Strongy Detect_new is an IgG—detecting ELISA based on the recombinant antigens NIE and SsRI [14, 15] Compared with the previous version, a cutoff to define positivity is available in the technical sheet (previously it was recommended to calculate it with a ROC curve based on the results also of other tests applied to the same individuals), and an unspecified number of components have been changed (personal communication).

The aim of this study was to evaluate the sensitivity and specificity of the new version of the Strongy Detect ELISA (“Strongy Detect_new” from now on in this manuscript) using DBS collected in the ESTRELLA study, and compare them to the performance of the older version of the assay (“Strongy Detect” from now on in this manuscript).

Blood samples from finger prick collected in the field in Ecuador on filter paper, as described previously, and stored at room temperature at the Universidad Central de Ecuador (Quito, Ecuador), were used in this study. In order to test for IgG antibodies against *Strongyloides stercoralis*, DBS were eluted as described previously and the Strongy Detect_new kit was used according to manufacturer instructions. Briefly, 100 µl of each diluted DBS eluate was added to the appropriate ELISA plate well and incubated at 37 °C for 30 min. Then, ELISA plate was washed six times with 300 µl of washing buffer each, added 100 µl of horseradish peroxidase conjugate with mouse anti-human IgG and incubated 30 min at 37 °C. Plate was washed six times with 300 µl of washing buffer each and 100 µl of substrate 3, 3’, 5, 5’tetramethylbenzidine (TMB) and hydrogen peroxide in a citric-acid citrate buffer (pH 3.3–3.8) were added, incubated in dark at room temperature during 10 min and 50 µl of stop solution were added and absorbance at 450 nm was read. For determination of positives, it is recommended to calculate the ratio of the test sample optical density (OD) to the mean Cut-off Control value for the assay. Ratios ≥ 1.1 indicate positive samples, ratio ≤ 0.9 are negatives, and any samples in the range of 0.9 < ISR value < 1.1 is considered borderline and should be retested in duplicate to confirm results.

In the absence of a perfect reference standard for *S. stercoralis* infection, diagnostic accuracy of the evaluated tests was estimated using Bayesian latent class analysis (BLCA). This approach allows simultaneous estimation of test sensitivity, specificity, and infection prevalence by modelling the unobserved (latent) infection status of each individual, without assuming any test to represent a gold standard [16]. The BLCA framework used in this study follows the latent class model previously described in the ESTRELLA study [13]. Posterior estimates of sensitivity (Se), specificity (Sp), and prevalence were obtained through Markov Chain Monte Carlo (MCMC) sampling. Bayesian estimates are reported as posterior medians with corresponding 95% credible intervals (CrI).

Positive and negative predictive values (PPV and NPV) were derived from the posterior distributions of Se and Sp. Predictive values were calculated under three epidemiological scenarios (7%, 15%, and 30%). The prevalence of 7% corresponds to the weighted prevalence estimated in the present dataset, reflecting the population structure of the study area.

Agreement between the older and the new Strongy Detect ELISA assays was evaluated using both frequentist and Bayesian approaches. In the frequentist framework, Cohen’s kappa statistic was calculated with its 95% confidence interval. In the Bayesian framework, total agreement (probability of concordant results), positive agreement and negative agreement were estimated. Positive agreement was defined as the probability of concordant positive results relative to all situations in which at least one test is positive, and negative agreement as the probability of concordant negative results relative to all situations in which at least one test is negative [17]. These indexes, together with a Bayesian analogue of Cohen’s kappa, were estimated by computing them for each MCMC draw from the posterior distributions of sensitivity, specificity, and prevalence, from which the joint probabilities of concordant and discordant test results were derived [18], thereby incorporating uncertainty in test performance parameters and prevalence.

Direct comparison of the two ELISA tests was performed by estimating the posterior distributions of the differences in sensitivity and specificity (ΔSe = Se_new–Se_old; ΔSp = Sp_new–Sp_old). The posterior probability expressed the probability that the new test outperformed the previous version. Posterior probability values range from 0 to 1, with 0 completely disproving the hypothesis, and 1 confirming it with absolute certainty.

All analyses were performed in R (version 4.5.1). Posterior distributions were obtained via MCMC sampling implemented in JAGS software [19] through the runjags package in R [20].

Among the samples tested with the new assay, only one had a borderline result (ISR = 1.03). The sample was therefore re-tested, eventually resulting weakly positive (ISR = 1.11).

The Strongy Detect_new assay showed lower sensitivity than the older version: 66.7% (95% CrI 52.8–80.6) versus 78.8% (95% CrI 66.7–89.2), respectively. The posterior probability was 0.1, meaning that there is low (10%) chance that the sensitivity of the new assay is higher than that of the old one. Specificity was instead confirmed higher for the new version compared to the previous assay: 98.8% (95% CrI 97.9–99.4) versus 90.1% (95% CrI 87.6–92.4), respectively, posterior probability = 1. The agreement between the two versions of the assay was classified as fair according to the frequentist Cohen’s kappa statistic (0.38; 95% CI 0.26–0.49). In the Bayesian agreement analysis, the probability of concordant positive results increased with increasing prevalence scenarios, reflecting the greater proportion of infected individuals expected in higher-prevalence settings. Conversely, the probability of concordant negative results decreased as prevalence increased. As a consequence, Bayesian kappa values also increased with prevalence, reflecting the change in the balance between concordant positive and concordant negative results across prevalence scenarios (Table 1).

**Table 1:** Agreement between the two versions of the Strongy Detect ELISA, at different prevalence values

Metric	Prevalence		
	7%	15%	30%
Total agreement	87.0% (84.5–89.2)	84.6% (82.1–87.0)	80.2% (77.3–83.5)
Positive agreement	36.7% (29.2–44.6)	50.8% (41.8–59.6)	61.4% (51.8–70.4)
Negative agreement	92.8% (91.3–94.0)	90.9% (89.3–92.3)	86.7% (84.9–88.6)
Bayesian kappa	0.31 (0.23–0.39)	0.43 (0.33–0.52)	0.49 (0.38–0.59)

Estimated medians and 95% credible intervals are shown in parentheses

The sensitivity and specificity estimate of all tests applied on the original study population in Ecuador, recalculated after inclusion of the Strongy Detect_new assay results, are reported in Table 2. Positive and negative predictive values (PPV and NPV, respectively) for individual tests and for test combinations under different prevalence scenarios are reported in Fig. 1.

**Table 2:** Sensitivity and specificity of all tests and test combinations used in the ESTRELLA study

Test	Sensitivity	Specificity
RDT	80.5% (73.8–87.1)	91.9% (89.9–93.9)
Baermann	52.4% (40.7–65.5)	98.9% (98.1–99.5)
PCR	56.4% (45.0–69.8)	98.9% (98.0–99.5)
Bordier ELISA	63.3% (49.2–77.2)	98.8% (98.0–99.5)
Strongy Detect ELISA	78.8% (66.7–89.2)	90.1% (87.6–92.4)
Strongy Detect _new ELISA	66.7% (52.8–80.6)	98.8% (97.9–99.4)
PCR + Strongy Detect ELISA	91.0% (84.4–95.7)	89.0% (86.4–91.3)
PCR + Strongy Detect_new ELISA	85.8% (77.2–92.8)	97.6% (96.3–98.5)
Baermann + Strongy Detect ELISA	90.1% (83.0–95.2)	89.0% (86.4–91.3)
Baermann + Strongy Detect_new ELISA	84.4% (75.5–91.8)	97.6% (96.3–98.5)

Estimated medians and 95% credible intervals are shown in parentheses

**Fig. 1:**
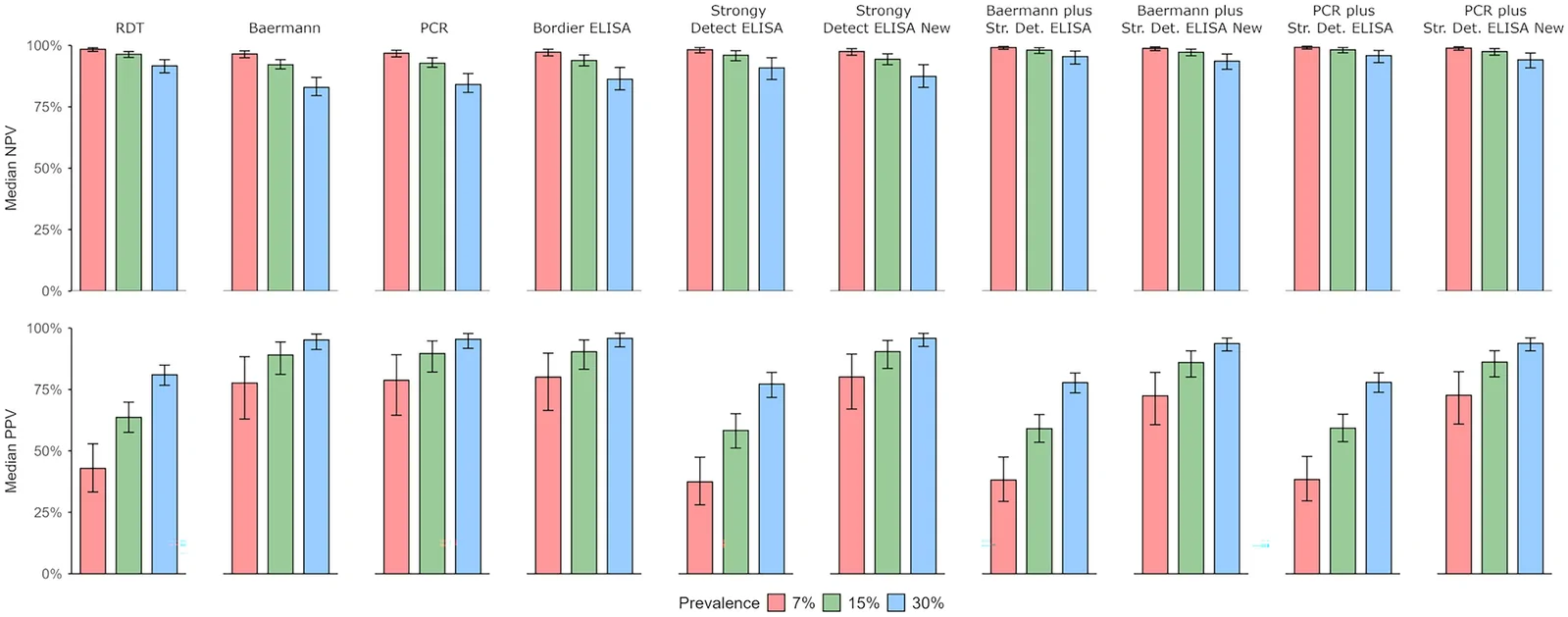
PPV and NPV for all tests and test combinations, at different prevalence scenarios (7%, 15% and 30%)

The new test demonstrated improved specificity and good sensitivity, with figures in line with the TPP for *S. stercoralis* diagnostics, which indicated ≥ 50% sensitivity and ≥ 98% specificity as the parameters for an ideal test to be used for control programmes [10, 11]. As expected, the combination with other diagnostic tests (i.e. Baermann, PCR) improved sensitivity, with a slight decrease in specificity. However, in the public health context excellent specificity is critical, while sensitivity values can be more relaxed [10]; implementation of a test combination would therefore likely not be worth as it would unnecessarily increase costs. The combination might instead be valuable for individual diagnosis, in a clinical setting.

The PPVs of the new assay were excellent at all the prevalence levels considered, being very close to those of the fecal tests; also, the ELISA based on *S. ratti* had almost identical PPVs. This finding may lead to questioning the higher prevalence threshold set by the WHO guideline for a serology test to identify areas where MDA is recommended, compared to fecal tests (15% of positive serology versus 5% of positive Baermann or agar plate culture in school age children) [1, 2].

Of note, there are several other parameters considered in the TPP, including ease of use, costs, shipping and storage conditions. The complete adherence of the new assay to all TPP requirements is out of the scope of the present study [11].

The main study limitation is that the testing conditions of the new assay differed from those of the previous version, as the DBS had been stored for a few years at room temperature in this study. However, we believe that this may have had limited or no impact on the estimation of the sensitivity of the Strongy Detect ELISA_new [21, 22]. Among the strengths, we highlight the wide panel of tests used to estimate the sensitivity and specificity and the use of the Bayesian Latent Class Analysis to estimate performance parameters in the absence of a gold standard for infection status classification.

In conclusion, the novel version of the Strongy Detect ELISA had a moderate reduction in sensitivity, while specificity improved compared to the previous version, overall meeting the requirements of the TPP released by the WHO. Additional parameters should be evaluated to confirm complete adherence to the TPP, thus suitability for deployment in control programmes for *S. stercoralis*.

## Data Availability

The raw data are available in Zenodo: https://zenodo.org/records/20792332.
